# Effect of ticagrelor combined with metoprolol extended-release tablets on cardiac function and clinical prognosis in elderly patients with acute coronary syndrome after percutaneous coronary intervention

**DOI:** 10.3389/fcvm.2025.1492569

**Published:** 2025-01-28

**Authors:** Lili Wang, Linlin Gao, Qin Chen, Li Chen, Hui Xu, Ling Sun, Youbin Hu

**Affiliations:** ^1^ Department of Cardiovascular Disease, Taizhou Jiangyan Hospital of Traditional Chinese Medicine, Taizhou, Jiangsu, China; ^2^ Department of Orthopedics, Taizhou Jiangyan Hospital of Traditional Chinese Medicine, Taizhou, Jiangsu, China

**Keywords:** ticagrelor, metoprolol extended-release tablets, acute coronary syndrome, percutaneous coronary intervention, cardiac function, clinical prognosis

## Abstract

**Background:**

Acute coronary syndrome (ACS) poses significant risks to older individuals. This study sought to assess the impact of combining ticagrelor and metoprolol extended-release tablets on clinical prognosis and cardiac function in elderly ACS patients following percutaneous coronary intervention (PCI).

**Methods:**

From February 2022 to February 2023, 90 elderly ACS patients who underwent PCI at our institution were retrospectively enrolled and divided into two groups: an observation group (OG) and a control group (CG), with 45 patients in each group. The CG received oral metoprolol extended-release tablets, while the OG received both oral metoprolol extended-release tablets and ticagrelor. Prognostic indicators and cardiac function were evaluated before and after treatment.

**Results:**

The treatment effectiveness rate in the OG was 97.78%, significantly higher than the CG's rate of 77.78% (*P* < 0.05). Post-treatment, the OG displayed notable improvements in cardiac function, including significantly higher left ventricular ejection fraction (LVEF), stroke volume (SV), cardiac output (CO), and cardiac index (CI) compared to the CG (*P* < 0.05). Both groups experienced enhanced exercise capacity, as evidenced by longer exercise duration (ED) and improved 6-min walking test (6MWT) results, with the OG showing superior gains (*P* < 0.05). Additionally, the OG had significantly higher serum levels of cardiac troponin T (cTnT) and creatine kinase isoenzyme (CK-MB) than the CG (*P* < 0.05). Decreases in serum levels of sICAM-1, MMP-9, and hs-CRP were observed in both groups, with more pronounced improvements in the OG (*P* < 0.05). The incidence of adverse prognostic events in the OG was significantly lower at 8.89%, compared to 37.78% in the CG (*P* < 0.05).

**Conclusion:**

Ticagrelor combined with metoprolol extended-release tablets can significantly improve cardiac function, motor performance, and quality of life in ACS patients after PCI. Additionally, it effectively increases myocardial injury markers and reduces serum inflammatory factor levels.

## 1 Introduction

Acute coronary syndrome (ACS) is a cardiovascular condition caused by the rupture or erosion of coronary atherosclerotic plaques, leading to partial or complete obstruction of the artery by a thrombus. This condition significantly increases global mortality and morbidity. ACS is considered a severe form of coronary artery disease, encompassing acute ST-segment elevation myocardial infarction (STEMI), unstable angina (UA) pectoris, and non-ST segment elevation myocardial infarction (NSTEMI) (1, 2). Without timely and appropriate treatment, ACS can lead to shock or sudden death, posing a serious threat to the patient's life (3–5). The main therapeutic option for ACS is percutaneous coronary intervention (PCI), which helps clear stenotic or occluded coronary arteries, improving myocardial perfusion and reducing the risk for coronary heart disease. However, stent placement and mechanical expansion during PCI can damage vascular cells, leading to complications such as bleeding and inflammation. Therefore, careful attention must be given to antiplatelet therapy. Once administered, antiplatelet agents inhibit platelet aggregation and adhesion, thereby reducing thrombosis at the site of vascular injury (6). Antithrombotic therapy is administered not only after PCI but also as part of the standard treatment for ACS, as platelets play a central role in the pathogenesis of ACS. Antithrombotic therapy can also avoid further injury of endothelium and cardiomyocytes, which is beneficial for repairing and protecting blood vessels and myocardium (7).

Inflammatory response, platelet activation, and vascular endothelial cell injury after Percutaneous coronary intervention (PCI) can lead to adverse cardiovascular events in patients with ACS. Metoprolol extended-release tablets, a *β*-receptor blocker, competitively and reversibly bind to *β*-adrenoceptors, reducing sympathetic activity, myocardial oxygen consumption, and plasma catecholamine levels. It also prolongs the diastolic period, improves coronary blood supply, inhibits ventricular remodeling, and improves cardiac function, reducing the risk of major adverse cardiac events (MACEs). However, its long-term effects are less favorable, and patients often relapse after discontinuing the drug (8, 9).. Recent studies have shown that low-density lipoprotein (LDL) cholesterol and oxidized LDL are significant risk factors for unstable angina in coronary heart disease. These lipoproteins can also promote the migration of leukocytes to the arterial intima, leading to endothelial cell damage (10, 11).

Ticagrelor is a novel antiplatelet medication that belongs to non-thiophene pyridine, which can act on platelet receptors directly without liver activation after oral administration and can effectively regulate lipids by blocking platelet receptors (12, 13). Additionally, ticagrelor benefits from a quick onset of action, stable pharmacological effects, and significant platelet inhibition in clinical settings (14). Since both ticagrelor and extended-release metoprolol have demonstrated efficacy for treating ACS individually, some experts and scholars have hypothesized that their combination could further enhance clinical outcomes. However, there is limited literature on the combined use of these treatments for managing patients undergoing PCI who develop ACS. Therefore, further research is needed to determine their potential value. The current study aims to evaluate the therapeutic efficacy, impact on cardiac function, and clinical prognosis of administering this dual treatment after PCI in elderly individuals with ACS. The findings will contribute to the body of knowledge and provide valuable insights for clinical practitioners.

## 2 Materials and methods

### 2.1 General information

**Figure 1:**
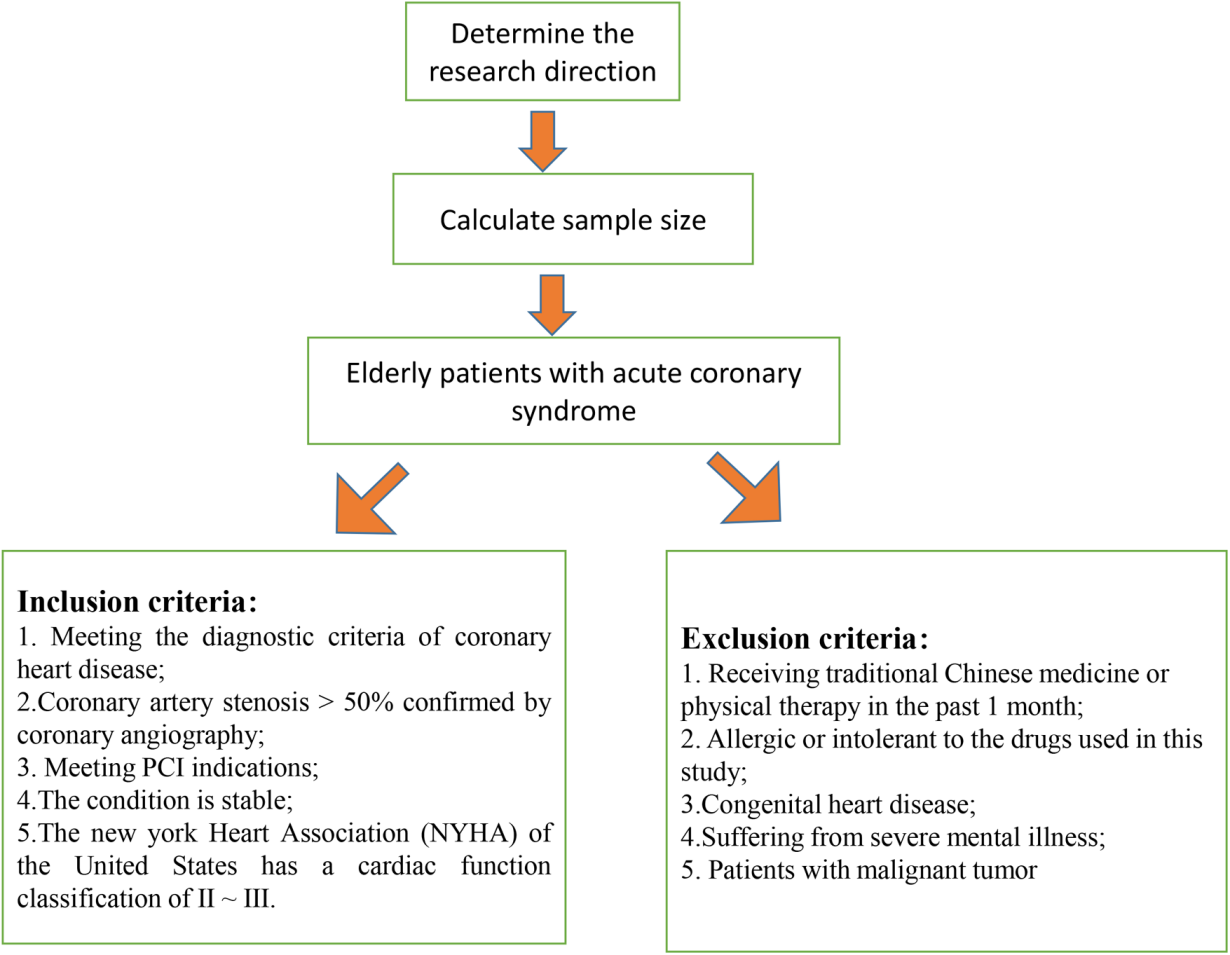
Schematic diagram of screening subjects into groups.

**Figure 2:**
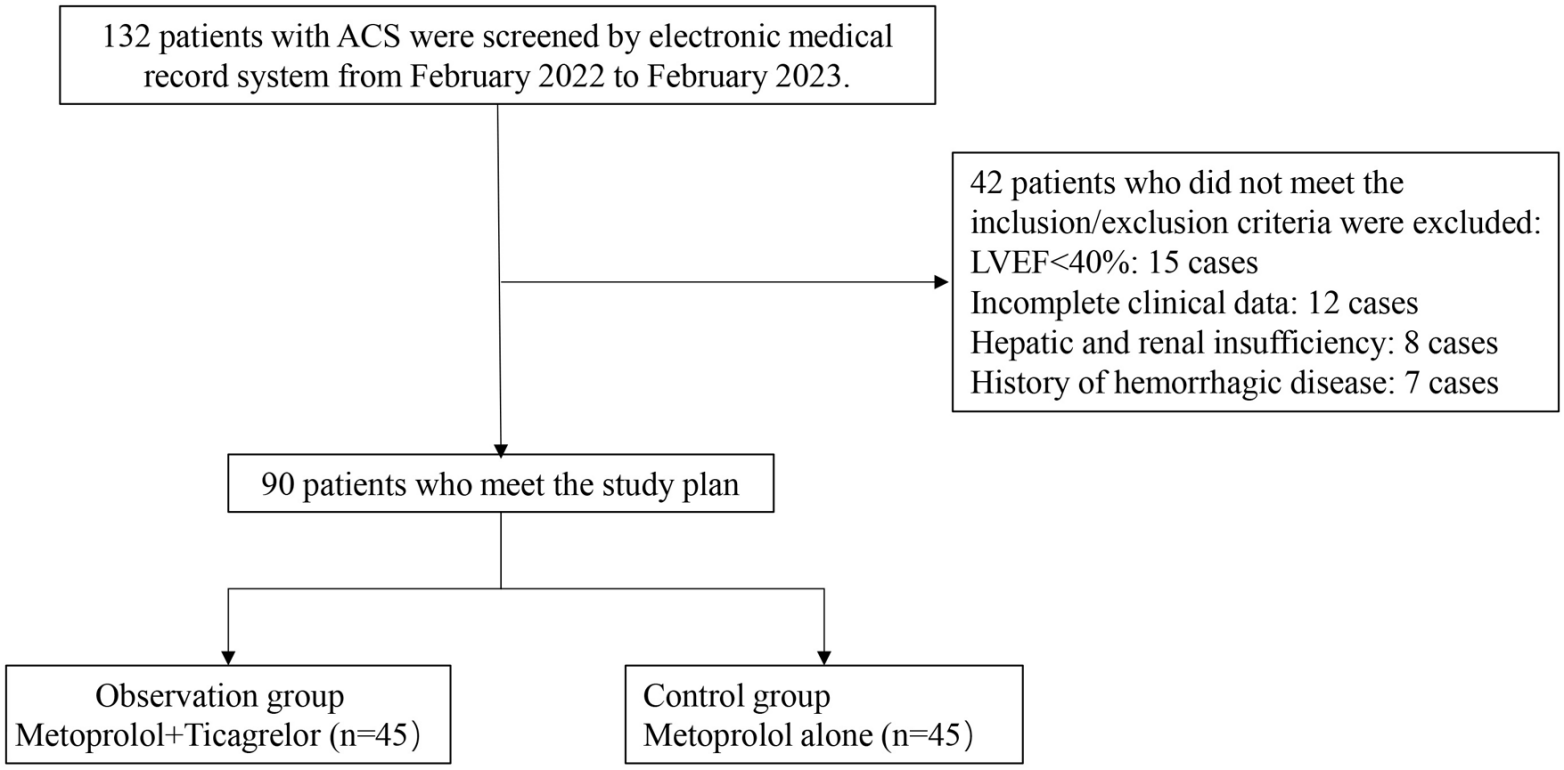
Flowchart depicting the patient selection process.

Between February 2022 and February 2023, we screened 142 patients diagnosed with ACS who underwent PCI at our institution for inclusion in this retrospective study. Figure 1 presents a schematic diagram illustrating the screening process of subjects into groups. The diagnosis of ACS in this study was based on the criteria outlined in the American Heart Association (AHA)/American College of Cardiology (ACC) guidelines (15). After applying the inclusion and exclusion criteria, a total of 90 elderly patients with a diagnosis of ACS were enrolled in the study. Patients were divided into two groups: an observation group (OG) and a control group (CG), with 45 patients in each group. The flowchart of patient selection process is illustrated in Figure 2. The division into groups was based on the treatment received at the time of admission, with the CG receiving oral metoprolol extended-release tablets and the OG receiving ticagrelor in addition to metoprolol extended-release tablets. Due to the nature of the retrospective design, the physicians involved in the treatment were aware of the treatment modalities prescribed to the patients, as treatment decisions were made based on clinical judgment and standard care protocols. The demographic and clinical characteristics of the patients were collected from medical records to ensure comprehensive data analysis. The study received approval from the institutional review boards of Taizhou Jiangyan Hospital of Traditional Chinese Medicine (reference number: RJ-2022-19) and was conducted in line with the Declaration of Helsinki principles. Because the study was retrospective and observational, and the data were anonymized, the requirement for informed consent was waived.

Inclusion criteria were as follows: (a) Patients aged 65 years or older who met the diagnostic criteria for ACS as outlined by the AHA/ACC guidelines, including those with STEMI, NSTEMI, and UA subtypes (15); (b) Coronary artery stenosis was confirmed by coronary angiography with >50% stenosis; (c) Patients with good cardiac function, indicated by a left ventricular ejection fraction (LVEF) of ≥40%, and no history of hemorrhagic diseases, allergies to hematopoietic agents, or significant complications, as well as no severe liver or kidney dysfunction, coagulation disorders, or active major hemorrhage; (d) The patient's condition was stable; (e) Patients were classified as Grade II to III according to the American New York Heart Association (NYHA) classification (16). Exclusion criteria were as follows: (a) Patients who had received traditional Chinese medicine or physiotherapy in the past month; (b) Individuals with allergies or intolerance to the medications used in this study; (c) Patients with congenital heart disease; (d) Individuals with severe mental illness; (e) Patients with malignant tumors; (f) Patients who required oral anticoagulants for comorbid conditions, such as atrial fibrillation or other thromboembolic disorders. We used the following formula to calculate the sample size:

$$n_{1}=\frac{{\left[Z_{\alpha /2}\sqrt{\;p(1-p)(1+c)/c+Z_{\beta }\sqrt{\;p_{1}(1-p_{1})+p_{2}(1-p_{2})/c}}\right]}^{2}}{{\left(\;p_{1}-p_{2}\right)}^{2}}$$

Bilateral *α* was set at 0.05 and *β* at 0.20. The clinical efficacy (total effective rate) was used as the effect index, based on relevant literature and previous studies (17), with P_1_ = 0.95 and P_2_ = 0.75. According to the calculation, 41 cases were needed in each group. Considering a dropout rate of 10%, approximately 45 patients were required per group, resulting in a total of 90 patients.

### 2.2 Treatment methods

Both groups in this study underwent PCI and received aspirin (100 mg once daily) as part of their standard post-PCI treatment regimen. Patients requiring oral anticoagulation for atrial fibrillation were excluded from the study. The CG was additionally treated with oral metoprolol extended-release tablets (AstraZeneca Pharmaceutical Company, Chinese Medicine Registration Number H37023121, batch numbers: 201552933, 201642358) at a dosage of 50 mg twice daily. The OG received a combination therapy of oral metoprolol extended-release tablets, administered at the same dosage as in the CG (50 mg twice daily), and ticagrelor, administered orally at a dose of 90 mg twice daily. Dual antiplatelet therapy (DAPT) was not used in the CG, as the aim was to evaluate the impact of adding ticagrelor to standard metoprolol therapy in the intervention group. This approach allowed us to assess the impact of a more potent antiplatelet regimen compared to a conservative, aspirin-only strategy. DAPT was initiated in the OG according to current ACS management guidelines, ensuring that all eligible patients received both ticagrelor and aspirin unless contraindications were present. The rationale for selecting ticagrelor centered on its demonstrated superiority over clopidogrel in reducing thrombotic events, particularly in high-risk ACS patients. We considered the feasibility of ticagrelor use by carefully evaluating patient characteristics, including age, comorbidities, and bleeding risk. The safety and appropriateness of administering ticagrelor were ensured by excluding patients with significant bleeding risks or contraindications. Patients with known contraindications to ticagrelor, such as a history of hemorrhagic stroke, active bleeding, or hypersensitivity to the drug, were excluded. A thorough pre-enrollment screening process was conducted to identify and exclude such patients, maintaining the safety and integrity of the study. Both groups were observed over a four-week period to assess the impact of the treatment regimens on cardiac function and clinical outcomes.

### 2.3 Outcome measure

The primary outcome was the treatment effectiveness rate, defined as a significant improvement in cardiac function and exercise capacity. Secondary outcomes included measurements of cardiac function (left ventricular ejection fraction [LVEF], stroke volume [SV], cardiac output [CO], and cardiac index [CI]), exercise performance [exercise duration <ed > and 6-minute walking test (6MWT)], and serum biomarker levels (cardiac troponin T [cTnT], creatine kinase isoenzyme [CK-MB], soluble intercellular adhesion molecule-1 [sICAM-1], matrix metalloproteinase-9 [MMP-9], and high-sensitivity C-reactive protein [hs-CRP]). The incidence of adverse prognostic events was also recorded.

### 2.4 Observation index

#### 2.4.1 Comparison of the clinical effectiveness between the two groups

The clinical efficacy of both groups was assessed after 4 weeks of therapy using the following evaluation criteria (17): (a) Significant Effect: ECG returned to normal or significantly improved, all symptoms disappeared, and there was an 80% reduction in cases of angina pectoris; (b) Effective: ECG and clinical symptoms improved, with a 50%–79% reduction in the occurrence of angina pectoris; (c) Invalid: Cases that did not meet the above criteria. The total effective rate is calculated as: (significant effect + effective cases)/total cases × 100%.

#### 2.4.2 Cardiac function assessment

Echocardiographic examinations were performed before treatment and 4 weeks after treatment by two experienced sonographers independently (*κ* coefficient >0.8). A GE Vivid E95 ultrasound system equipped with an M5Sc-D probe (1.4–4.6 MHz) was used. Measurements included left ventricular ejection fraction (LVEF), stroke volume (SV), cardiac output (CO), and cardiac index (CI). All measurements were averaged over three cardiac cycles.

#### 2.4.3 Exercise capacity assessment

Exercise capacity was evaluated using the six-minute walk test (6MWT) and exercise duration (ED) before and 4 weeks after treatment. The 6MWT was conducted in a standard 30-meter hospital corridor, recording the total walking distance covered within 6 min. Heart rate and oxygen saturation were monitored throughout the test. The test was terminated if the heart rate exceeded 85% of maximum, oxygen saturation fell below 90%, or patients experienced severe fatigue or chest pain.

#### 2.4.4 Biomarker detection

Biomarker measurements were conducted before treatment and 4 weeks after treatment. Blood samples for these markers were collected at baseline (prior to treatment initiation) and again at the end of the 4-week treatment period to evaluate changes over time. These included myocardial injury markers such as cardiac troponin T (cTnT) and creatine kinase-MB isoenzyme (CK-MB), as well as inflammatory factors like soluble intercellular adhesion molecule-1 (sICAM-1), matrix metalloproteinase-9 (MMP-9), and high-sensitivity C-reactive protein (hs-CRP). Blood samples were processed within 30 min of collection by centrifugation (3,000 rpm for 15 min), and the separated serum was stored at −80°C until analysis. All test kits were purchased from Shanghai JieYi Biotechnology Co., Ltd. (Shanghai, China) and were used strictly according to the manufacturer's instructions.

#### 2.4.5 Quality of life assessment

Quality of life (18) was evaluated before treatment and 4 weeks after treatment using a quality-of-life questionnaire that assessed daily living function, psychological function, social function, and material living conditions. Each dimension was scored on a scale of 0–100 points, with higher scores indicating better quality of life in that dimension. Assessments were conducted by trained nurses through face-to-face interviews.

#### 2.4.6 Prognosis

The incidence of adverse prognostic events, including angina pectoris, myocardial infarction, in-stent thrombosis, and death, was recorded in both treatment groups. The total incidence was calculated by dividing the total number of adverse events by the total number of cases and multiplying by 100%. Angina pectoris is characterized by paroxysmal, compressive chest pain, often accompanied by other symptoms (19). Myocardial infarction refers to the sudden necrosis of the myocardium caused by prolonged ischemia and hypoxia of the coronary artery, with chest pain as the primary symptom (20). In-stent thrombosis occurs when the endothelium is damaged, exposing subendothelial tissue after stent implantation (21). It can be triggered by stent rupture or the development of new atherosclerotic plaque within the stent, leading to rapid platelet aggregation and thrombus formation. Symptoms may include chest pain, chest tightness, and dyspnea.

### 2.5 Statistical analysis

The data were analyzed using the statistical software SPSS 22.0. Measurement data with a normal distribution and homogeneous variance were expressed as mean ± standard deviation (x¯ ± s). Independent sample *t*-tests were used to compare data between groups, while paired *t*-tests were performed within each group. Categorical data, expressed as *n* (%), were analyzed using the chi-square (*χ*^2^) test. A *P*-value of less than 0.05 was considered statistically significant.

## 3 Results

### 3.1 Baseline characteristics of the study population

The CG consisted of 22 females and 23 males, with an age range of 65–83 years and an average age of 72.71 ± 5.62 years. Regarding vessel involvement, 25 cases had single-vessel involvement, while 20 cases had double-vessel involvement. The average body mass index (BMI) was 22.87 ± 2.31 kg/m^2^, ranging from 17.88 to 27.74 kg/m^2^. Regarding education level, 20 participants had completed primary or junior high school, 10 had completed junior college or higher education, and 15 had graduated from senior high school or technical secondary school. The OG comprised 19 females and 26 males, with an average age of 73.89 ± 5.81 years, ranging from 65 to 83 years. Among the OG participants, 26 cases had single-vessel involvement, while 19 cases had double-vessel involvement. BMI values ranged from 17.90 to 27.80 kg/m^2^. The education level distribution was as follows: 18 participants had completed primary or junior middle school, 11 had completed junior college or higher education, and 16 had graduated from senior high school or technical secondary school. There were no significant differences between the CG and OG in terms of gender distribution, age, vessel involvement, BMI, or education level (*p* > 0.05 for all comparisons) (Supplementary Table S1).

### 3.2 Comparison of clinical efficacy between the two groups

**Figure 3:**
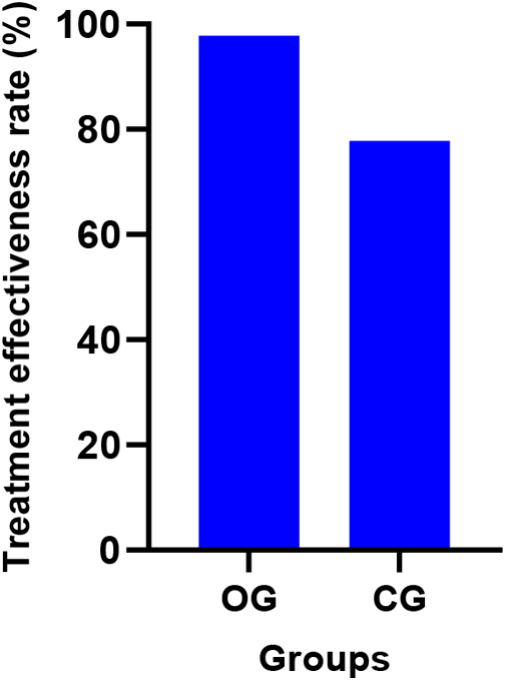
Comparison of clinical efficacy between the two groups (%). OG, Observation group; CG, Control group.

The treatment effectiveness rate in the OG was 97.78% (44/45 patients), which was significantly higher than the CG's rate of 77.78% (35/45 patients) (*P* < 0.05). Figure 3 displays the effectiveness rates for both groups.

### 3.3 Comparison of cardiac function indexes between the two groups before and after treatment

Before therapy, there were no discernible differences in cardiac function between the two groups (*P* > 0.05). After therapy, cardiac function indexes, including LVEF, SV, CO, and CI, were significantly higher in the observation group compared to the CG (*P* < 0.05). All outcomes are presented in detail in Table 1.

**Table 1:** Comparison of cardiac function indexes between the two groups before and after treatment ($\bar{x}$±s, *n* = 45).

Group	LVEF (%)		SV (ml)		CO (L/min)		CI (min·m^2^)	
	Before treatment	After treatment	Before treatment	After treatment	Before treatment	After treatment	Before treatment	After treatment
OG	44.12 ± 4.58	59.88 ± 5.06^a^	51.05 ± 5.82	68.83 ± 6.42^a^	3.41 ± 0.82	5.83 ± 1.05^a^	2.67 ± 0.58	3.92 ± 0.67^a^
CG	44.03 ± 4.19	53.24 ± 4.91^b^	50.89 ± 5.44	62.41 ± 5.28^b^	3.52 ± 0.59	5.04 ± 0.81^b^	2.83 ± 0.44	3.22 ± 0.55^b^
*t*	0.097	6.317	0.135	5.181	0.730	3.996	1.474	5.417
*P*	0.923	<0.001	0.893	<0.001	0.467	<0.001	0.149	<0.001

Values are presented as Mean ± SD. Compared with the OG before treatment.

OG, observation group; CG, control group.

^a^ *P* < 0.05; compared with the CG before treatment.

^b^ *P* < 0.05.

### 3.4 Comparison of motor assessment scale between the two groups before and after treatment

The two groups had no significant difference in baseline 6MWT and ED scores (*P* > 0.05). After therapy, the 6MWT and ED scores significantly improved in both groups, with the OG showing a notably better improvement than the CG (*P* < 0.05). All outcomes are detailed in Table 2.

**Table 2:** Comparison of motor function indexes between the two groups before and after treatment (¯x ± s, *n* = 45).

Group	6MWT (m)		ED (s)	
	Before treatment	After treatment	Before treatment	After treatment
OG	381.39 ± 44.23	409.52 ± 45.66^a^	360.49 ± 27.04	379.28 ± 27.09^a^
CG	380.84 ± 44.09	390.04 ± 44.21^b^	360.43 ± 26.55	365.05 ± 27.14^b^
*t*	0.059	2.056	0.007	2.489
*P*	0.953	0.045	0.995	0.018

Values are presented as Mean ± SD. Compared with the OG before treatment.

OG, observation group; CG, control group.

^a^ *P* < 0.05; compared with the CG before treatment.

^b^ *P* < 0.05.

### 3.5 Comparison of the levels of myocardial injury-related factors between the two groups before and after treatment

**Figure 4:**
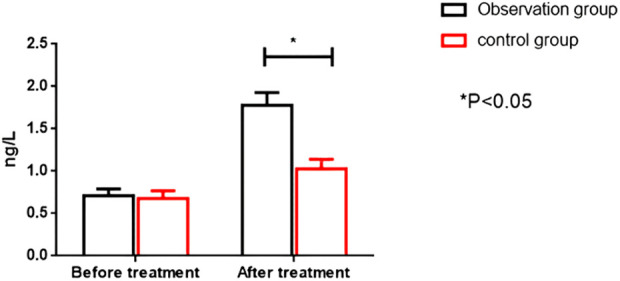
Comparison of CK-MB levels between the two groups before and after intervention. CK-MB; Creatine kinase MB isoform.

**Figure 5:**
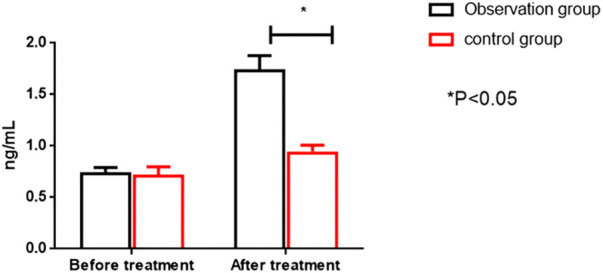
Comparison of cTnT levels between the two groups before and after intervention. cTnT; cardiac troponin I.

Before therapy, the two groups had no significant differences in serum CK-MB and cTnT levels (*P* > 0.05). After treatment, the OG showed significantly higher serum CK-MB and cTnT levels than the CG (*P* < 0.05). All results are presented in Figures 4, 5.

### 3.6 Comparison of serum levels of inflammatory factors between the two groups before and after treatment

Before therapy, the two groups had no significant differences in the concentrations of sICAM-1, MMP-9, and hs-CRP (*P* > 0.05). After treatment, serum levels of sICAM-1, MMP-9, and hs-CRP decreased in both groups, with the OG showing a significantly greater improvement than the CG (*P* < 0.05). All results are detailed in Table 3.

**Table 3:** Comparison of serum indexes between the two groups before and after treatment ($\bar{x}$ ±s, *n* = 45).

Group	sICAM-1 (ng/ml)		MMP-9 (*μ*g/L)		hs-CRP	
	Before treatment	After treatment	Before treatment	After treatment	Before treatment	After treatment
OG	645.53 ± 109.91	439.91 ± 87.04^a^	182.42 ± 20.38	90.45 ± 11.34^a^	26.18 ± 5.03	18.81 ± 4.02^a^
CG	644.15 ± 115.03	347.22 ± 65.68^b^	181.94 ± 21.34	62.17 ± 7.53^b^	26.54 ± 5.19	13.15 ± 3.24^b^
*t*	0.058	5.702	0.109	13.936	0.334	7.354
*P*	0.954	<0.001	0.914	<0.001	0.740	<0.001

Values are presented as Mean ± SD. Compared with the OG before treatment.

OG, observation group; CG, control group.

^a^ *P* < 0.05; compared with the CG before treatment.

^b^ *P* < 0.05.

### 3.7 Comparison of quality of life scores between the two groups

Before therapy, there was no statistically significant difference between the two groups regarding daily, psychological, social, and material functioning (*P* > 0.05). After the intervention, scores for quality of life across all categories significantly improved in both groups, with the OG showing a more significant improvement than the CG (*P* < 0.05). The results are detailed in Table 4.

**Table 4:** Comparison of life quality scores between the two groups (points, $\bar{x}$ ±s).

Group	Daily life function		Psychological function		Social function		Material function	
	Before treatment	After treatment	Before treatment	After treatment	Before treatment	After treatment	Before treatment	After treatment
OG	32.24 ± 5.12	60.22 ± 9.14^a^	21.82 ± 2.41	67.48 ± 8.15^a^	30.46 ± 4.84	66.82 ± 7.11^a^	22.82 ± 8.12	69.67 ± 8.37^a^
CG	31.23 ± 5.07	47.31 ± 7.15^b^	21.13 ± 2.09	56.33 ± 8.24^b^	30.17 ± 4.78	52.51 ± 4.72^b^	22.71 ± 8.06	55.91 ± 8.23^b^
*t*	0.94	7.463	1.451	6.454	0.293	11.248	0.064	7.864
*P*	0.351	<0.001	0.152	<0.001	0.761	<0.001	0.949	<0.001

Values are presented as Mea*n* ± SD. Compared with the OG before treatment.

OG, observation group; CG, control group.

^a^ *P* < 0.05; compared with the CG before treatment.

^b^ *P* < 0.05.

### 3.8 Comparison of adverse prognostic events between the two groups

The incidence of adverse prognostic events was significantly lower in the OG (8.89%) compared to the CG (37.78%) (*P* < 0.05). The detailed results of adverse prognostic events between the two groups are shown in Table 5.

**Table 5:** Comparison of the incidence of adverse prognostic events involving both groups (n/%).

Group	*N*	Angina	Myocardial infarction	In-stent thrombus	Death	Total incidence rate（%）
OG	45	1（2.22）	2（4.44）	0（0.00）	1（2.22）	4（8.89）
CG	45	3（6.67）	5（11.11）	6（13.33）	3（6.67）	17（37.78）
*χ2*						10.497
*P*						0.003

Values are presented as count (percentage).

OG, observation group; CG, control group.

## 4 Discussion

In recent years, the number of ACS cases has been rising as the aging population in China increases. The clinical treatment of ACS is based on the principles of restoring coronary blood flow, improving myocardial oxygen consumption, alleviating myocardial ischemia, and preventing coronary thrombosis to reduce complications and mortality (22). For elderly patients with ACS, it is difficult to tolerate revascularization due to declining body functions and organ dysfunction, making drug therapy the preferred choice.

ACS is primarily caused by intracoronary thrombosis, which leads to coronary artery occlusion and myocardial injury. Thrombosis occurs when the fibrous cap of an atherosclerotic plaque ruptures, releasing a highly thrombotic lipid core into the bloodstream. This triggers a series of signaling pathways that activate platelets, initiating the coagulation cascade and promoting thrombosis (23). Therefore, in conventional treatment, alongside coronary artery dilation and the use of angiotensin receptor blockers (ARBs) and angiotensin-converting enzyme inhibitors (ACE inhibitors), anticoagulant and antiplatelet therapies play a significant role in managing ACS and are integral to the treatment process. However, long-term use of anticoagulants and antiplatelet agents increases the risk of bleeding. Furthermore, elderly patients with ACS often have multiple underlying conditions, and the adverse effects of prolonged medication can negatively impact adherence to the prescribed treatment regimen. As a result, the effectiveness of conventional treatment is often suboptimal due to various complex factors. Hence, there is an urgent need to identify a treatment for elderly ACS patients that is both effective and safe (24, 25).

Ticagrelor is a novel antiplatelet medication that can quickly and effectively inhibit adenosine diphosphate-mediated platelet aggregation, significantly reducing the incidence of adverse events such as myocardial infarction and cardiovascular death (26, 27). In the present study, the efficacy rate in the OG was 97.78%, which was statistically higher than that of the CG (77.78%). After treatment, cardiac function indicators such as LVEF, SV, CO, and CI were significantly higher in the OG compared to the CG, and both the 6MWT and ED were longer in the OG. These results suggest that metoprolol extended-release tablets can improve cardiac and motor functions, thereby enhancing the therapeutic effect. However, the therapeutic effect of combining metoprolol with ticagrelor was even better. Ticagrelor is a novel oral antiplatelet medication known for its potent antiplatelet effects. It works by inhibiting the P2Y12 receptor on platelets, binding to it reversibly, and effectively reducing platelet aggregation. This mechanism of action leads to significant anticoagulant effects, improving myocardial blood supply and enhancing coronary artery blood flow (28). Metoprolol extended-release tablets are widely used due to their rapid absorption, fast peak time, and short plasma half-life. In contrast, succinic acid, which has lower solubility than tartaric acid, allows for the sustained and controlled release of the drug over an extended period. This prolonged release leads to stable blood concentrations with minimal fluctuations between peak and trough levels (29). Additionally, metoprolol extended-release tablets utilize a multi-unit microcapsule-controlled release technique that ensures a slow and consistent release. The absorption process lasts over 20 h, resulting in a longer plasma half-life. Taken once daily, the tablets maintain stable 24-h blood concentrations, provide ideal *β*1-receptor blockade, enhance drug compliance, and improve cardiac function in patients (30). The combination of these two drugs can have a synergistic effect, further enhancing therapeutic outcomes.

While the myocardial infarction with non-obstructed coronary arteries (MINOCA) study (31) reported a high risk of adverse events during follow-up, especially reinfarction, with nearly half of the patients with re-AMI experiencing progression of atherosclerosis, our study found that PCI combined with dual antiplatelet therapy resulted in a lower rate of reinfarction, which may be attributed to more aggressive management strategies. Inflammation is a key factor influencing the prognosis of ACS patients, as it is strongly associated with adverse cardiovascular outcomes. Research on STEMI showed that elevated T2 values in the non-infarcted myocardium were correlated with larger infarct sizes, microvascular obstruction, and left ventricular dysfunction, emphasizing the role of inflammation at the tissue level (32). These findings suggest that inflammation, especially within the non-infarcted myocardium, could serve as an important predictor of reinfarction and other major adverse cardiac events in ACS patients. Previous research has shown that unstable coronary artery disease-related angina pectoris is associated with a low level of chronic inflammation in the body, and the inflammatory response within plaques is a key factor contributing to plaque instability (33). Adipose tissue in the body secretes various bioactive substances, including the immunoglobulin superfamily member sICAM-1, which is expressed and secreted by smooth muscle cells and endothelial cells in atherosclerotic plaques. sICAM-1 is currently recognized as an independent risk factor for predicting ACS. Previous literature has also indicated that elevated levels of MMPs serve as a separate risk factor for the progression of ACS (34). This increase is closely associated with plaque instability and can be used as a diagnostic marker for ACS, as well as for assessing the extent of infarction and predicting prognosis. Hepatocytes produce the acute phase protein hs-CRP, which can promote thrombosis and is a risk factor for unstable angina pectoris. Its serum level plays a crucial role in ACS's intervention and prognosis assessment. This research demonstrated that after therapy, serum levels of sICAM-1, MMP-9, and hs-CRP decreased in both groups, with the improvement in the OG being significantly better than in the CG. These results suggest that ticagrelor may more effectively inhibit the release of inflammatory factors. Mechanistically, metoprolol extended-release tablets can enhance the oxygen supply to myocardial cells, improve the aerobic metabolism of the myocardium, reduce the production of free fatty acids, and alleviate angina pectoris symptoms. Ticagrelor reversibly inhibits platelet aggregation, but it is effective only after biotransformation into active metabolites. To improve patient prognosis, excessive doses may lead to medication resistance. When myocardial injury occurs, serum levels of CK-MB and cTnT rise significantly, making CK-MB and cTnT commonly used indicators for diagnosing and predicting the prognosis of ACS. After treatment, the OG's serum CK-MB and cTnT levels were higher than those of the CG, with this difference being statistically significant. It is hypothesized that treating elderly ACS patients with a combination of ticagrelor and metoprolol extended-release tablets is beneficial. This combination may reduce inflammatory and oxidative stress responses, improve immune indexes, prevent cardiomyocyte damage, and enhance therapeutic outcomes. According to our findings, the quality of life scores in the OG were higher than those in the CG. This is because the combination of ticagrelor with metoprolol extended-release tablets was more effective than metoprolol extended-release tablets alone. This combination better improves the patient's condition and helps them return to their regular lives more quickly. The rate of adverse prognostic events was lower in the OG compared to the CG. The daily blood concentration of metoprolol extended-release tablets remains within the therapeutic window, selectively blocking *β*1 receptors while avoiding adverse effects associated with *β*2 receptors.

There are several limitations to this study. First, as a retrospective observational analysis, we cannot eliminate the potential for bias and confounding factors. Second, being a single-center study limits its generalizability, and the small sample size may affect the reliability of the findings. Third, a limitation of the study is that the CG received only aspirin and metoprolol without DAPT. This conservative approach, in line with current ACS guidelines (35), was intentionally chosen to assess the added benefit of ticagrelor in the intervention group OG. Lastly, the dosages of ticagrelor and metoprolol used in this study are not universally recommended and may differ from standard guidelines in various regions. Future prospective studies involving multiple centers are needed to validate these findings.

## 5 Conclusion

In summary, using a combination of ticagrelor and metoprolol extended-release tablets after PCI can enhance the overall effectiveness and cardiac function in patients with coronary heart disease and reduce the occurrence of adverse prognostic events, compared to metoprolol extended-release tablets alone. However, prospective multi-center studies with larger sample sizes are required to validate our findings.

## Data Availability

The raw data supporting the conclusions of this article will be made available by the authors, without undue reservation.
