# Supplementary material for: Effect of ticagrelor combined with metoprolol extended-release tablets on cardiac function and clinical prognosis in elderly patients with acute coronary syndrome after percutaneous coronary intervention
Source: Front Cardiovasc Med. 2025 Jan 28;12:1492569. doi: 10.3389/fcvm.2025.1492569 (PMC11810974; doi:10.3389/fcvm.2025.1492569)
Supplement: Supplementary file 1 [file Table1.docx]

**Supplementary Table 1. Baseline Characteristics of the Control Group and Observation Group**

| **Characteristic** | **Control Group (CG) (n=45)** | **Observation Group (OG)**  **(n=45)** | **p-value** |
| --- | --- | --- | --- |
| Gender (Male) | 23 (51.11%) | 26 (57.78%) | 0.451 |
| Age (years) | 72.71 ± 5.62 | 73.89 ± 5.81 | 0.462 |
| **Vessel Involvement** |  |  |  |
| Single | 25 (55.56) | 26 (57.78) | 0.834 |
| Double | 20 (44.44) | 19 (42.22) | 0.884 |
| BMI (kg/m²) | 22.87 ± 2.31 | 22.76 ± 2.45 | 0.753 |
| **Education Level** |  |  | 0.912 |
| Primary/Junior High | 20 (44.44%) | 18 (40.00%) |  |
| Junior College/Above | 10 (22.22%) | 11 (24.44%) |  |
| Senior High/University | 15 (33.33%) | 16 (35.56%) |  |

Note: Values are presented as Mean ± SD or count (percentages).
